# Efficacy of topical 0.05% cyclosporine A and 0.1% sodium hyaluronate in post-refractive surgery chronic dry eye patients with ocular pain

**DOI:** 10.1186/s12886-024-03294-z

**Published:** 2024-01-22

**Authors:** Lu Zhao, Jiawei Chen, Hongyu Duan, Tingting Yang, Baikai Ma, Yifan Zhou, LinBo Bian, Xiying Cai, Hong Qi

**Affiliations:** 1 Department of Ophthalmology, Beijing Key Laboratory of Restoration of Damaged Ocular Nerve, Peking University Third Hospital, Beijing, China; 2 Institute of Medical Technology, Peking University Health Science Center, 49 North Garden Road, Haidian District, Beijing, 100191 China; 3 Department of Ophthalmology, Guangdong Provincial People’s Hospital (Guangdong Academy of Medical Sciences, Southern Medical University, Guangzhou, China; 4 Peking University First Hospital, Beijing, China

**Keywords:** Post-refractive surgery dry eye disease, Ocular pain, Cyclosporine A, Sodium hyaluronate, Ocular inflammation, Tear film stability

## Abstract

**Background:**

The management of post-refractive surgery dry eye disease (DED) can be challenging in clinical practice, and patients usually show an incomplete response to traditional artificial tears, especially when it is complicated with ocular pain. Therefore, we aim to investigate the efficacy of combined topical 0.05% cyclosporine A and 0.1% sodium hyaluronate treatment in post-refractive surgery DED patients with ocular pain unresponsive to traditional artificial tears.

**Methods:**

We enrolled 30 patients with post-refractive surgery DED with ocular pain who were unresponsive to traditional artificial tears. Topical 0.05% cyclosporine A and 0.1% sodium hyaluronate were used for 3 months. They were evaluated at baseline and 1 and 3 months for dry eye and ocular pain symptoms and objective parameters, including Numerical Rating Scale (NRS), Neuropathic Pain Symptom Inventory modified for the Eye (NPSI-Eye), tear break-up time (TBUT), Schirmer I test (SIt), corneal fluorescein staining (CFS), corneal sensitivity, and corneal nerve morphology. In addition, tear levels of inflammatory cytokines and neuropeptides were measured using the Luminex assay.

**Results:**

After 3 months of treatment, patients showed a statistically significant improvement in the ocular surface disease index (OSDI), TBUT, SIt, CFS, and corneal sensitivity (all *P* < 0.01) using linear mixed models. As for ocular pain parameters, the NRS and NPSI-Eye scores were significantly reduced (both *P* < 0.05) and positively correlated with the OSDI and CFS scores. Additionally, tear IL-1β, IL-6, and TNF-α levels were improved better than pre-treatment (*P* = 0.01, 0.03, 0.02, respectively).

**Conclusion:**

In patients with post-refractive surgery DED with ocular pain, combined topical 0.05% cyclosporine A and 0.1% sodium hyaluronate treatment improved tear film stability, dry eye discomfort, and ocular pain, effectively controlling ocular inflammation.

**Trial registration:**

Registration number: NCT06043908.

## Background

Nowadays, corneal refractive surgery offers a choice of procedures, such as laser-assisted in situ keratomileusis (LASIK), femtosecond laser-assisted laser in situ keratomileusis (FS-LASIK), and small-incision lenticule extraction (SMILE), all of which are associated with high indices of efficacy and safety. Nonetheless, dry eye disease (DED) is the most common complication after corneal refractive surgery and one of the leading causes of patient dissatisfaction [1–4]. Although DED generally occurs transiently in the early postoperative period, it may also develop into a chronic condition, and approximately 18–41% of patients develop chronic DED for more than 6 months [4–7]. It becomes more worrisome when combined with ocular pain.

Conventional artificial tears, such as sodium hyaluronate, are the first-line therapy for patients with DED and temporarily alleviate dry eye symptoms owing to their water-retentive properties [8, 9]. However, its therapeutic mechanism is single; it may not be sufficient enough to treat DED following refractive surgery [10]. In addition, patients with DED combined with ocular pain were more likely to show an incomplete response to conventional artificial tears than those without ocular pain. Anti-inflammatory drugs, such as topical glucocorticoids and cyclosporine, are recommended for patients who are unresponsive to treatment with conventional artificial tears [11]. However, long-term use of topical glucocorticoids can lead to complications such as steroid-induced glaucoma and cataracts [12].

Cyclosporine is an immunosuppressive agent widely used to treat various autoimmune diseases and has been approved by the United States Food and Drug Administration (FDA) for treating DED [13]. In a prospective study, 0.05% cyclosporine improved the symptoms and signs of patients with DED, with significant differences compared to conventional artificial tears [14]. However, the aforementioned studies did not evaluate the effects of cyclosporine on ocular pain, corneal nerves, tear cytokines, and neuropeptides, which are also involved in the mechanism of post-refractive surgery DED, especially ocular pain. Neurotrophic inflammation caused by corneal nerve damage has been suggested as a causative factor for this type of DED [15]. The DEWS report suggested that anti-inflammatory therapy plays an essential role in maintaining ocular surface homeostasis [11]. The efficacy of cyclosporine and sodium hyaluronate have been evaluated before; however, it is not clear whether the combination therapy of these can be helpful for patients with post-refractive surgery DED with ocular pain. This study aimed to evaluate the combined effect of topical 0.05% cyclosporine A and 0.1% sodium hyaluronate treatment on post-refractive surgery DED associated with ocular pain that was not responsive to conventional artificial tears.

## Methods

This prospective study aimed to investigate the effects of combination therapy with 0.05% cyclosporine A and 0.1% sodium hyaluronate eye drops in post-refractive surgery dry eye patients with ocular pain. The study patients used 0.05% cyclosporine A eyedrops (GYZZ H20203239, Shenyang Xingqi Pharmaceutical Co Ltd.) twice a day and 0.1% sodium hyaluronate eyedrops (HyloComod®, Ursapharm, Saarbrucken, Germany) four times a day for 3 months. Dry eye and ocular pain symptoms, ocular surface parameters, corneal nerve, tear cytokines, and neuropeptides were measured at baseline and at 1-month and 3-month visits after commencing treatment. This study was approved by the Ethics Committee of the Peking University Third Hospital and followed the principles of the Declaration of Helsinki. Written informed consent was obtained from all participants.

To determine the appropriate sample size for this study, we utilized PASS 15.0 software for power analysis. The calculation was based on changes observed in OSDI scores among dry eye patients treated with 0.05% cyclosporine eye drops in a previous study by Shin D and Sang Min J [16]. In this reference study, the baseline OSDI score was 25.30 ± 19.04, which significantly decreased to 13.63 ± 14.94 after three months of treatment (*P* < 0.001). With an alpha level set at 0.05 and a power of 0.9, the analysis indicated a necessity for a minimum of 25 participants. Considering an anticipated dropout rate of 10%, we aimed to enroll 28 participants. Ultimately, our study included 30 participants, thus satisfying and marginally exceeding the calculated sample size requirement.

## Participants

The inclusion criteria were as follows: (1) diagnosed with DED [17] (ocular surface disease index [OSDI] score ≥ 13 and tear breakup time [TBUT] < 10 s) continuing for at least 6 months after corneal refractive surgery [7]; (2) experienced ocular pain, which was indicated by a Numerical Rating Scale (NRS) score ≥ 2 [18]; (3) nonresponsive to artificial tear treatment for more than 3 months based on both symptoms and signs; (4) patients were able to follow up for at least three months; (5) All patients underwent a comprehensive dry eye evaluation prior to refractive surgery, with no patients being diagnosed with preoperative DED. Participants were excluded if they had active ocular disease, anti-inflammatory therapy, other previous ocular surgery, or other major systemic diseases, including malignant tumors and autoimmune diseases. Pregnant and nursing mothers were excluded from the study.

## Ocular surface evaluations

The OSDI questionnaire (0–100) evaluated DED-related symptoms, and the overall score classified patients into the following groups: 0–12, absence of symptoms; 13–22, mild symptoms; 23–32, moderate symptoms; and 33–100, severe symptoms [19, 20]. Ocular pain severity was assessed using the NRS questionnaire, which rated the pain intensity as none (0), mild (1–3), moderate (4–6), or severe (7–10) [21]. Ocular pain characteristics were described using the Neuropathic Pain Symptom Inventory modified for the Eye (NPSI-Eye; range 0–100) [22].

We performed TBUT, Schirmer I test (SIt), corneal fluorescein staining (CFS), and conjunctival lissamine green (LG) staining to evaluate ocular surface signs. The measurements were performed from least to most invasively. Ocular surface assessments were performed in both eyes at all visits. The right eye was selected for the analysis. TBUT was evaluated using a cobalt blue filter over a slit-lamp biomicroscope. SIt was conducted using Schirmer paper strips (5 × 35 mm) without anesthesia. CFS and LG staining were evaluated using the National Eye Institute Workshop guidelines (total score:0–15) [23] and the Oxford grading panel (total score:0–10) [24], respectively.

The corneal sub-basal nerve plexus was imaged using an in vivo laser scanning confocal microscope (IVCM [Heidelberg Retinal Tomograph III with a Rostock Corneal Module; Heidelberg Engineering GmbH, Germany]). Five representative images of the sub-basal nerve plexus of the central cornea were selected for analysis (resolution:384 × 384 pixels; area: 400 × 400 mm [0.16 mm^2^]). The morphological parameters of the corneal nerves were analyzed using ACCMetrics software (University of Manchester, UK) [25]. The nerve parameters included corneal nerve fiber density (CNFD), corneal nerve branch density (CNBD), corneal nerve fiber length (CNFL), corneal nerve fiber total branch density (CTBD), and corneal nerve fiber width (CNFW).

Corneal sensitivity is one way to evaluate the function of corneal nerves and was measured using a Cochet-Bonnet esthesiometer (Luneau Ophthalmologie, Chartres Cedex, France) with a 6.0-cm adjustable nylon monofilament. Starting at 6.0 cm, the monofilament length was gradually reduced at 5-mm intervals until the initial response occurred.

## Analysis of tear cytokine and neuropeptide concentrations

Approximately 5 µl of the unstimulated basal tears from the right eye were collected from the lower tear meniscus with a clean glass micropipette (Microcaps; Drummond Scientific Co, Broomall, PA) in a reasonable time (up to 5 min) without provoking a reflex secretion of tears, and samples were stored at -80 °C as soon as possible. The levels of inflammatory cytokines (interferon [IFN]-γ, interleukin [IL]-10, IL-17 A, IL-1β, IL-23, IL-6 and tumor necrosis factor-α [TNF-α]) and neuropeptides (α-melanocyte-stimulating hormone [α-MSH], oxytocin, and substance P [SP]) were detected using the MILLIPLEX® Human High Sensitivity T Cell Magnetic Bead Panel (Millipore, Billerica, MA, USA) and MILLIPLEX MAP® Human Neuropeptide Magnetic Bead Panel (Millipore, Billerica, MA, USA), separately. All procedures were performed according to the manufacturer’s instructions [26].

## Statistical analyses

Statistical analyses were performed using SPSS software (version 27.0; SPSS Inc., Chicago, IL, USA). Figures were created using GraphPad Prism 9.4 software package and R software (version 4.3.1). The normality assumption was checked using the Shapiro–Wilk test. The variables are expressed as the mean ± standard deviation (SD) or medians (interquartile ranges) according to their distributions. Linear mixed models were used to assess changes in the studied variables over time. The Bonferroni adjustment was used for multiple comparisons. Spearman’s rank correlation was used to explore the relationship between ocular parameters. Statistical significance was set at *P* < 0.05.

## Results

### Participant demographics

In this study, a total of 30 participants were enrolled in the study, all of whom met the inclusion criteria and successfully completed the entire follow-up process. The participants’ characteristics are presented in Table 1. Among the 30 patients, 24 were women and 6 were men. The mean age was 34.40 ± 7.02. The mean preoperative spherical equivalent (SE) was − 5.30 ± 1.75D.

**Table 1:** The demographic data of participants

Basic information	
Participants/eyes - (n/n)	30/30
LASIK - n (%)	11 (36.7%)
FS-LASIK - n (%)	11 (36.7%)
SMILE - n (%)	8 (26.6%)
Female/male - (n/n)	24/6
Age (y) - mean (SD)	34.40 ± 7.02
Preoperative SE (D) - mean (SD)	-5.30 ± 1.75
Duration after RS (y) - median (Q1, Q3)	5 (1.88, 10.00)
Duration of dry eye after RS (y) - median (Q1, Q3)	2 (1.00, 8.00)
Duration of ocular pain after RS (y) - median (Q1, Q3)	0.5 (0.30, 6.00)

n, number; LASIK, laser-assisted in situ keratomileusis; FS-LASIK, femtosecond laser assisted laser in situ keratomileusis; SMILE, small-incision lenticule extraction; y, year; SD, standard deviation; SE, spherical equivalent; D, Diopter; RS, refractive surgery

### Ocular surface parameters

**Fig. 1:**
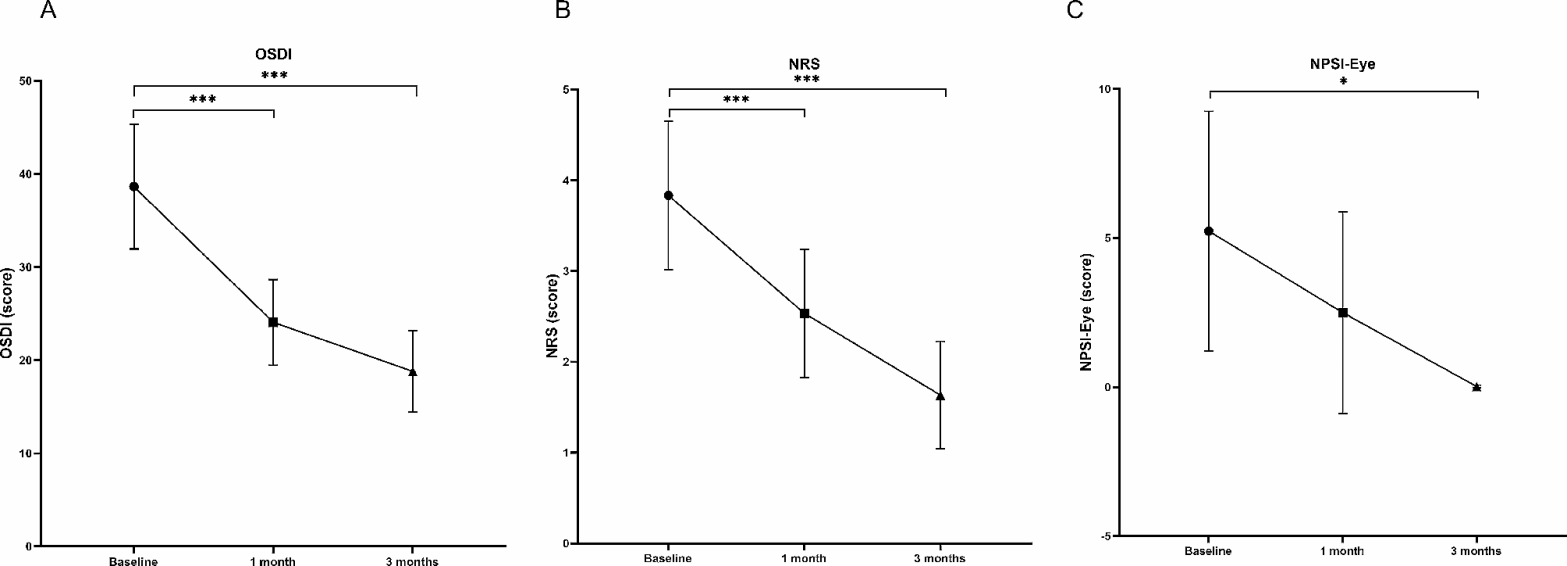
Changes in (**A**) OSDI scores, (**B**) NRS scores, and (**C**) NPSI-Eye scores at 1 and 3 month treatment compared with baseline values. Data is expressed as mean and 95% confidence interval. OSDI: Ocular Surface Disease Index; NRS: Numerical Rating Scale; NPSI-Eye: Neuropathic Pain Symptom Inventory modified for the Eye. *P<0.05; **P<0.01; ***P<0.001

**Fig. 2:**
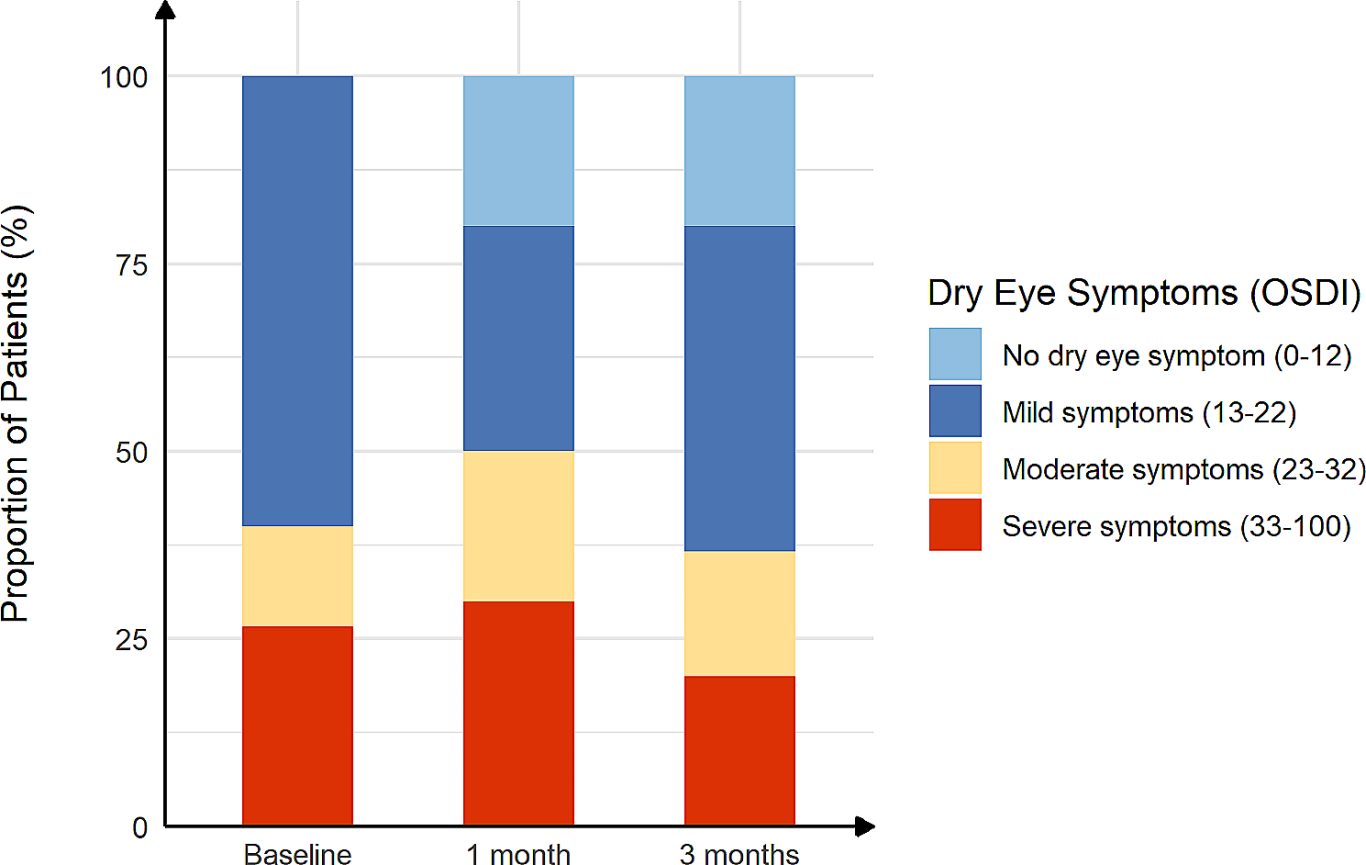
The proportion of severity of dry eye symptoms in post–refractive surgery DED patients with ocular pain before and after treatment. The severity of dry eye symptoms was scored according to Ocular Surface Disease Index (OSDI) (range, 0-100)

**Fig. 3:**
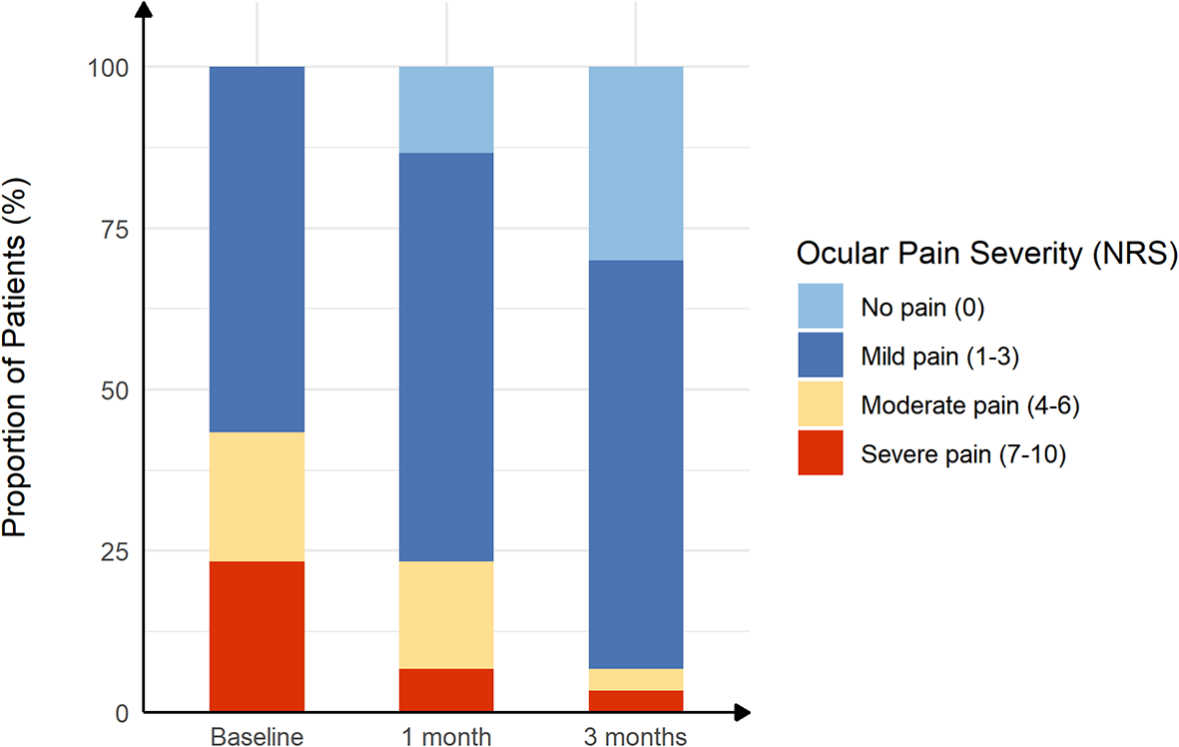
The proportion of ocular pain severity in post–refractive surgery DED patients with ocular pain before and after treatment. The severity of ocular pain was scored according to Numerical Rating Scale (NRS) (range, 0–10)

**Fig. 4:**
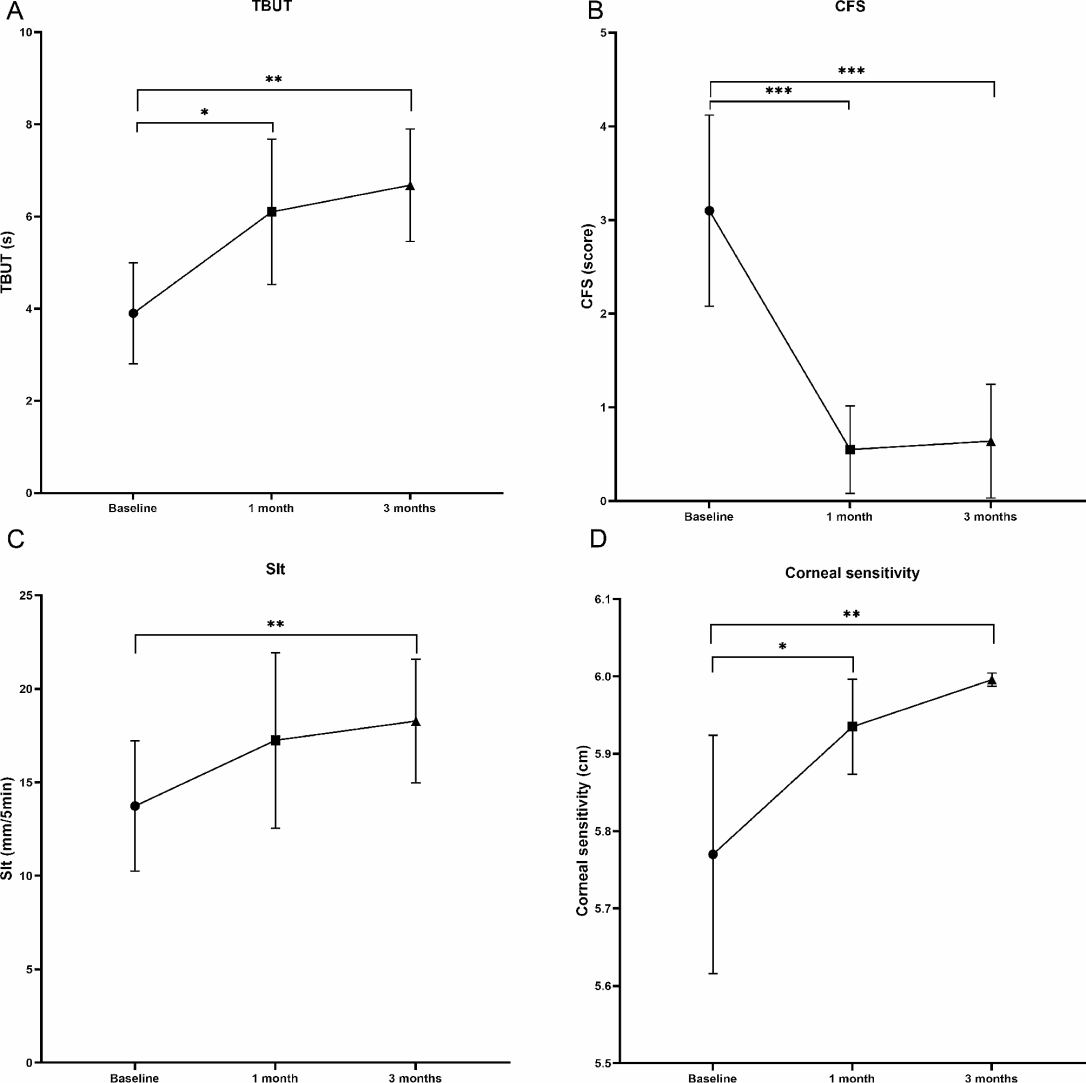
Changes in (**A**) TBUT scores, (**B**) CFS scores, (**C**) SIt scores, and (**D**) Corneal sensitivity at 1 and 3 month treatment compared with baseline values. Data is expressed as mean and 95% confidence interval. TBUT: tear break-up time; CFS: corneal fluorescein staining; SIt: Schirmer I test. *P<0.05; **P<0.01; ***P<0.001

The mean OSDI scores decreased from the pre-treatment (baseline) levels after 1 and 3 months of treatment (both *P* < 0.01) (Fig. 1). In addition, after 3 months of treatment, the proportion of mild, moderate, and severe dry eye symptoms changed from 60%, 13%, and 27%, respectively, to 43%, 17%, and 20%, respectively, and dry eye symptoms disappeared in 6 of 30 patients (20%) (Fig. 2). As expected, the NRS and NPSI-Eye scores significantly reduced after 3 months of treatment (both *P* < 0.05), especially for burning spontaneous pain (*P* = 0.04) and evoked pain (*P* = 0.03) (Table 2). Meanwhile, after 3 months treatment, the proportion of mild, moderate, and severe ocular pain changed from 57%, 20%, and 23%, respectively, to 63%, 3%, and 3%, respectively, and dry eye symptoms disappeared in 9 of 30 patients (30%) (Fig. 3). The mean TBUT and SIt scores increased significantly after 3 months of treatment compared to baseline (both *P* < 0.01) (Fig. 4). The CFS scores were significantly reduced after 1 and 3 month treatment compared to baseline (both *P* < 0.01) (Fig. 4). There were no significant differences in LG scores between pre-treatment and post-treatment (*P* = 0.11) (Table 2). In the correlation analysis, ocular pain scores were positively correlated with the OSDI and CFS scores and negatively correlated with the TBUT scores (Table 3).

**Table 2:** Ocular surface parameters of participants before and after treatment

Characteristics	Baseline	1 m	3 m	P-value
**Ocular symptoms**				
OSDI (score) - mean (SD)	38.65 ± 17.93	24.06 ± 12.33	18.80 ± 11.75	<0.01
Severe (33–100) - n (%)	8 (27%)	9 (30%)	6 (20%)	
Moderate (23–32) - n (%)	4 (13%)	6 (20%)	5 (17%)	
Mild (13–22) - n (%)	18 (60%)	9 (30%)	13 (43%)	
No dry eye symptom (0–12) - n (%)	0 (0%)	6 (20%)	6 (20%)	
NRS (score) - median (Q1, Q3)	3 (2, 5.5)	2 (2, 3.25)	2 (0, 2)	<0.01
Severe pain (7–10) - n (%)	7 (23%)	2 (7%)	1 (3%)	
Moderate pain (4–6) - n (%)	6 (20%)	5 (17%)	1 (3%)	
Mild pain (1–3) - n (%)	17 (57%)	19 (63%)	19 (63%)	
No pain (0) - n (%)	0 (0%)	4 (13%)	9 (30%)	
NPSI-Eye (score) - mean (SD)	5.23 ± 10.76	2.50 ± 7.23	0.02 ± 0.10	0.02
Burning spontaneous pain	0.83 ± 1.89	0.25 ± 0.71	0.02 ± 0.10	0.04
Pressing spontaneous pain	0.46 ± 1.09	0.33 ± 0.86	0.04 ± 0.20	0.05
Paroxysmal pain	0.11 ± 0.31	0.13 ± 0.56	0.02 ± 0.10	0.08
Evoked pain	0.79 ± 1.88	0.35 ± 1.08	0.05 ± 0.02	0.03
Paresthesia/dysesthesia	0.43 ± 1.25	0.15 ± 0.67	0.04 ± 0.20	0.21
**Ocular signs**				
TBUT (s) - mean (SD)	3.90 ± 2.92	6.10 ± 3.37	6.68 ± 2.95	<0.01
CFS (score) - median (Q1, Q3)	3.00 (0, 5)	0 (0, 1)	0 (0, 0.5)	<0.01
LG (score) - median (Q1, Q3)	0 (0, 2)	0 (0, 0)	0 (0, 0)	0.11
Schirmer I test (mm) - mean (SD)	13.73 ± 9.33	17.25 ± 10.02	18.28 ± 7.98	<0.01
**Corneal nerve parameters- mean (SD)**				
Corneal sensitivity (cm)	5.77 ± 0.41	5.93 ± 0.13	5.99 ± 0.02	<0.01
CNFD (n/mm^2^)	20.66 ± 9.14	20.90 ± 8.37	20.27 ± 7.85	0.76
CNBD (n/mm^2^)	46.83 ± 33.51	43.25 ± 31.16	39.13 ± 31.65	0.22
CNFL (mm/mm^2^)	15.93 ± 5.10	15.07 ± 4.66	15.06 ± 4.21	0.18
CTBD (n/mm^2^)	83.02 ± 53.32	70.62 ± 44.51	68.14 ± 46.25	0.15
CNFW (mm /mm^2^)	0.021 ± 0.00	0.022 ± 0.00	0.022 ± 0.00	0.69

OSDI: Ocular Surface Disease Index; NRS: Numerical Rating Scale; NPSI-Eye: Neuropathic Pain Symptom Inventory modified for the Eye; TBUT, tear break-up time; CFS, corneal fluorescein staining; LG: Lissamine green; CNFD, corneal nerve fiber density; CNBD, corneal nerve branch density; CNFL, corneal nerve fiber length; CTBD, corneal nerve fiber total branch density; CNFW, corneal nerve fiber width. SD, standard deviation; *P*<0.05 with mixed linear model

**Table 3:** Correlations between ocular symptoms and signs

	OSDI (score)	NRS (score)	NPSI (score)
OSDI (score)	*r* = 1	*r* = 0.375* *p*<0.001	*r* = 0.387* *p*<0.001
CFS (score)	*r* = 0.318* *p* = 0.005	*r* = 0.326* *p* = 0.004	*r* = 0.243* *p* = 0.037
TBUT (s)	*r* = -0.342* *p* = 0.003	*r* = -0.269* *p* = 0.020	*r* = -0.152 *p* = 0.195
Schirmer I test (mm)	*r* = 0.085 *p* = 0.467	*r* = 0.031 *p* = 0.789	*r* = 0.132 *p* = 0.263

The r and p values were determined with Spearman’s correlation coefficient. *, *P*<0.05

OSDI, Ocular Surface Disease Index; NRS, Numerical Rating Scale; NPSI-Eye, Neuropathic Pain Symptom Inventory modified for the Eye; CFS, corneal fluorescein staining; TBUT, tear break-up time

Corneal sensitivity significantly increased after 1 and 3 months of treatment compared to the pre-treatment values (*P* < 0.05) (Fig. 4). There were no significant differences in CNFD, CNBD, CNFL, CTBD, or CNFW at follow-up (all *P* > 0.05) (Table 2).

### Tear cytokine and neuropeptide concentrations

**Fig. 5:**
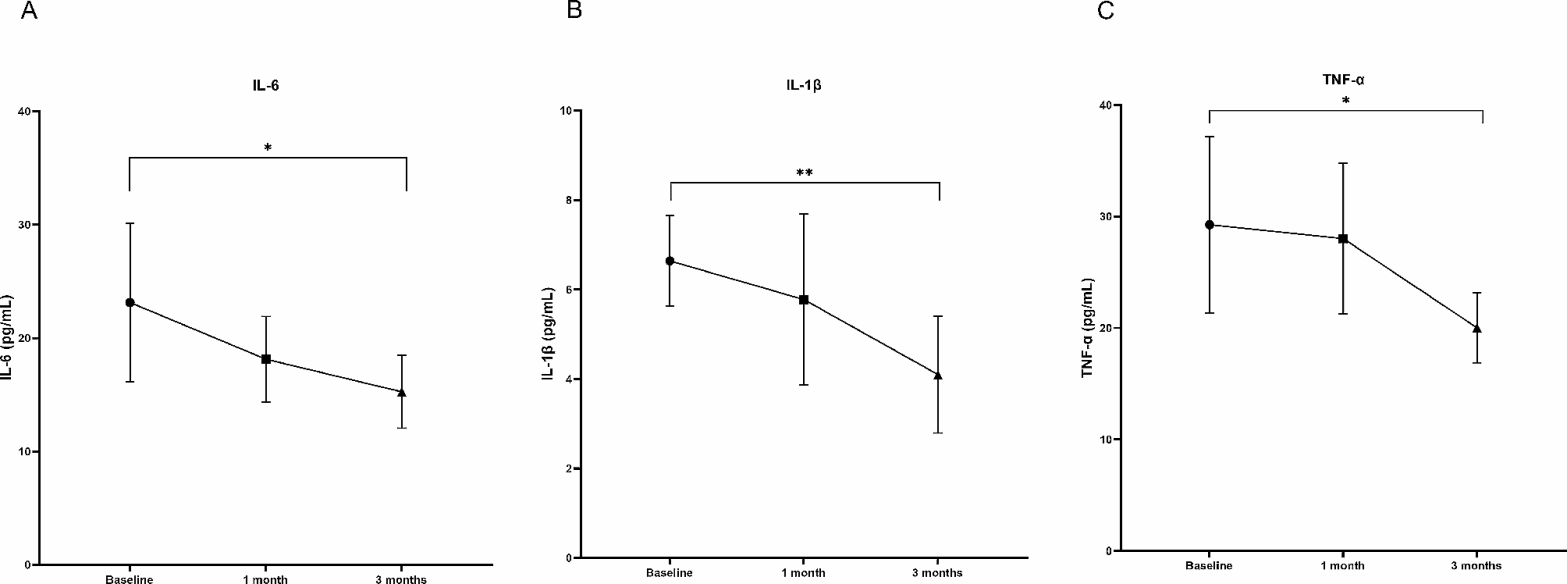
Changes in tear (**A**) IL-6, (**B**) IL-1β, (**C**) TNF-α levels after 1 and 3 month treatment compared with baseline values. Data is expressed as mean and 95% confidence interval. IL, interleukin; TNF-α, tumor necrosis factor-α. *P<0.05; **P<0.01

Tear inflammatory cytokine and neuropeptide concentrations in the participants before and after treatment are shown in Table 4. There was no significant difference in the concentrations of all inflammatory factors and neuropeptides before and after 1 month of treatment. After 3 month treatment, tear IL-6, IL-1β, TNF-α levels were decreased than baseline (*P* = 0.03; *P* = 0.01; *P* = 0.02, respectively) (Fig. 5). As for other inflammatory cytokines, including IL-10, IL-17 A, INF-γ, and IL-23, no statistical difference was found. There were no statistically significant differences in neuropeptide concentrations before and after treatment.

**Table 4:** Tear inflammatory cytokines and neuropeptide concentrations of participants before and after treatment

Concentrations (pg/ml)	Baseline	1 m	3 m	P-value	
IFN-γ - mean (SD)	41.91 ± 58.26	47.18 ± 26.85	43.87 ± 49.87	0.82	
IL-10 - mean (SD)	56.19 ± 61.57	60.65 ± 103.74	46.47 ± 32.82	0.18	
IL-17 A - mean (SD)	50.37 ± 86.65	52.62 ± 86.04	42.38 ± 26.14	0.59	
IL-1β- mean (SD)	6.64 ± 2.62	5.77 ± 4.64	4.10 ± 3.23	0.01	
IL-23 - mean (SD)	1653.00 ± 2452.30	1184.77 ± 1167.66	1027.63 ± 584.39	0.28	
IL-6 - mean (SD)	23.13 ± 18.08	18.14 ± 8.49	15.26 ± 7.08	0.03	
TNF-α- mean (SD)	29.26 ± 20.38	28.01 ± 17.42	20.02 ± 8.10	0.02	
α-MSH - mean (SD)	223039.65 ± 151360.67	257683.48 ± 148820.68	292800.05 ± 139057.84	0.11	
Oxytocin - mean (SD)	149426.33 ± 96328.34	155149.79 ± 74552.44	175864.65 ± 63998.12	0.37	
SP - mean (SD)	37922.01 ± 35813.01	40321.37 ± 19040.77	46294.62 ± 17023.66	0.24	

INF-γ, Interferon-γ; IL, interleukin; TNF-α, tumor necrosis factor-α; α-MSH, alpha-melanocyte stimulating hormone; SP, substance P; SD, standard deviation. *P*<0.05 with mixed linear model

## Discussion

DED is one of the most common complications associated with corneal refractive surgery. According to previous reports, DED affects approximately 85.4% of patients at 1 week postoperatively and 59.4% of patients at 1 month after refractive surgery [27, 28]. While DED usually occurs transiently in the early postoperative period, it could also develop into a chronic condition; approximately 8–20% of patients develop chronic DED for more than 6 months [4–6]. Traditional artificial tears are often poor or even ineffective in these patients. Additionally, a number of patients experience some form of ocular pain [29]. Owing to the lack of understanding of ocular pain, there is currently no effective drug for treating it, which substantially impacts the quality of life.

This is the first study to evaluate the therapeutic effects of 0.05% cyclosporine and sodium hyaluronate eye drops in patients with post-refractive surgery DED with ocular pain unresponsive to traditional artificial tears. Our results showed that the topical combined application of 0.05% cyclosporine A and sodium hyaluronate eye drops had beneficial effects on the relief of dry eye and ocular pain symptoms and on improving tear film stability and ocular inflammation.

Almost 80% of the participants in this study were women. This is consistent with previous findings showing that women are more likely to develop refractive surgery-related DED [30]. The results showed that in DED patients who are ineffective in treating sodium hyaluronate alone, the dry eye symptoms and ocular pain improved significantly after using cyclosporine combined with sodium hyaluronate for 3 months, especially burning spontaneous pain and evoked pain. Moreover, we observed a positive correlation between dry eye symptoms and ocular pain symptoms and a negative correlation between BUT scores and ocular pain symptoms, indicating that DED may cause ocular pain to some extent.

Cyclosporine is an agent reported to promote the secretion of aqueous tears. In this study, after 3 months of treatment, TBUT and SIt had significant improvements, especially at 3 months of treatment. The degree of corneal fluorescein staining was significantly lower than that before treatment, indicating that the corneal epithelium was repaired gradually. Moreover, we found that the ocular pain score positively correlated with the degree of corneal fluorescein staining, indicating that corneal epithelial injury was one of the factors causing ocular pain in these patients.

The cornea is densely innervated by sensory neurons that are responsible for corneal perception when the ocular surface is exposed to harmful stimuli or inflammation [31]. There were no significant differences in the morphology of the corneal subbasal nerves between pre-treatment and post-treatment. Interestingly, corneal perception was better than before treatment, consistent with a previous study by Toker and Asfuroğlu [32]. This may be due to the neurotrophic effect of cyclosporine, either by directly acting on nerve cells or by reestablishing a healthy environment for nerve regeneration [33]. However, improved corneal perception and nerve function did not show the same trend. This may be because corneal perception mainly represents the density of the subepithelial nerve endings and does not completely reflect the density and length of the corneal subbasal nerve.

Cyclosporine can regulate the underlying inflammatory pathology of the ocular surface by binding to cyclophilin in lymphocytes, blocking the expression of immune mediators such as IL-1β, IL-6, and interferon-γ [34]. In DED, hyperosmotic factors disturb the dynamic balance of the ocular surface, resulting in an imbalance between secretion and degradation of tear film components. Tear film instability increases the risk of corneal epithelial injury, which leads to the release of inflammatory mediators. Immune cells on the ocular surface release a large number of proinflammatory cytokines, which recruit more immune cells to accumulate on the ocular surface, leading to a vicious circle of inflammation [35]. This study showed a significant reduction in tear inflammatory cytokine levels at 3 months. Still, no difference was observed at 1 month, suggesting that cyclosporine has a slower but better effect. Short-term treatment limits its benefits; therefore, long-term treatment for at least 3 months is considered necessary.

Although sodium hyaluronate can improve dry eye symptoms to some extent, it fails to address the underlying cause of the disease, namely, inflammation. Consequently, their clinical efficacies are limited. Without adequate treatment, the ocular surface can become progressively damaged. Therefore, during the treatment of DED, especially post-refractive surgery DED, it is appropriate to improve the tear film while addressing the inflammatory response of the ocular surface [36].

This study has some limitations. One of these limitations was the short study duration. This is because cyclosporine eye drops are thought to inhibit the recruitment of T cells, but this process may take 3–6 months [37]. Despite the limited duration of this study, the results remain valid. Second, all patients received combination treatment, which may have confounded the interpretation of the effects of cyclosporine. However, the recruited patients did not respond to sodium hyaluronate treatment. Hence, the positive outcomes observed were unlikely to have been affected by the lubricants.

## Conclusions

In general, cyclosporine not only alleviates ocular pain and dry eye symptoms and signs in post-refractive surgery DED patients with ocular pain but also effectively controls ocular inflammation. Therefore, cyclosporine may be an effective alternative treatment for post-refractive surgery in patients with DED and ocular pain.

## Data Availability

All data generated or analyzed during this study are included in this publication. The relevant raw data will be freely available from the corresponding author upon request.

## Abbreviations

α-MSH: α-Melanocyte-Stimulating Hormone
CFS: Corneal Fluorescein Staining
CNBD: Corneal Nerve Branch Density
CNFD: Corneal Nerve Fiber Density
CNFL: Corneal Nerve Fiber Length
CTBD: Corneal Nerve Fiber Total Branch Density
FS-LASIK: Femtosecond Laser-Assisted Laser In Situ Keratomileusis
DED: Dry Eye Disease
LASIK: Laser-Assisted In Situ Keratomileusis
NRS: Numerical Rating Scale
CNFW: Corneal Nerve Fiber Width
OSDI: Ocular Surface Disease Index
SIt: Schirmer I Test Scores
SMILE: Small-Incision Lenticule Extraction
SP: Substance P
TBUT: Tear Break-Up Time
SE: Spherical Equivalent
FDA: United States Food And Drug Administration
TNF-α: Tumor Necrosis Factor-α
