# Correction: Efficacy of topical 0.05% cyclosporine A and 0.1% sodium hyaluronate in post-refractive surgery chronic dry eye patients with ocular pain

**DOI:** 10.1186/s12886-024-03471-0

**Published:** 2024-05-28

**Authors:** Lu Zhao, Jiawei Chen, Hongyu Duan, Tingting Yang, Baikai Ma, Yifan Zhou, LinBo Bian, Xiying Cai, Hong Qi

**Affiliations:** 1 Department of Ophthalmology, Beijing Key Laboratory of Restoration of Damaged Ocular Nerve, Peking University Third Hospital, Beijing, China; 2 Institute of Medical Technology, Peking University Health Science Center, 49 North Garden Road, Haidian District, Beijing, 100191 China; 3 Department of Ophthalmology, Guangdong Provincial People’s Hospital (Guangdong Academy of Medical Sciences, Southern Medical University, Guangzhou, China; 4 Peking University First Hospital, Beijing, China

**Correction: BMC Ophthalmol 24, 28 (2024)**

10.1186/s12886-024-03294-z

Following publication of the original article [1], the authors would like to replace Figure 3 as it mistakenly uses the bar graph from Figure 2.

Figure 2 remains accurate and unaffected.

This error does not impact the conclusions of the article.

The correct and incorrect version of Fig. 3 can be found below:

Correct

**Fig. 3:**
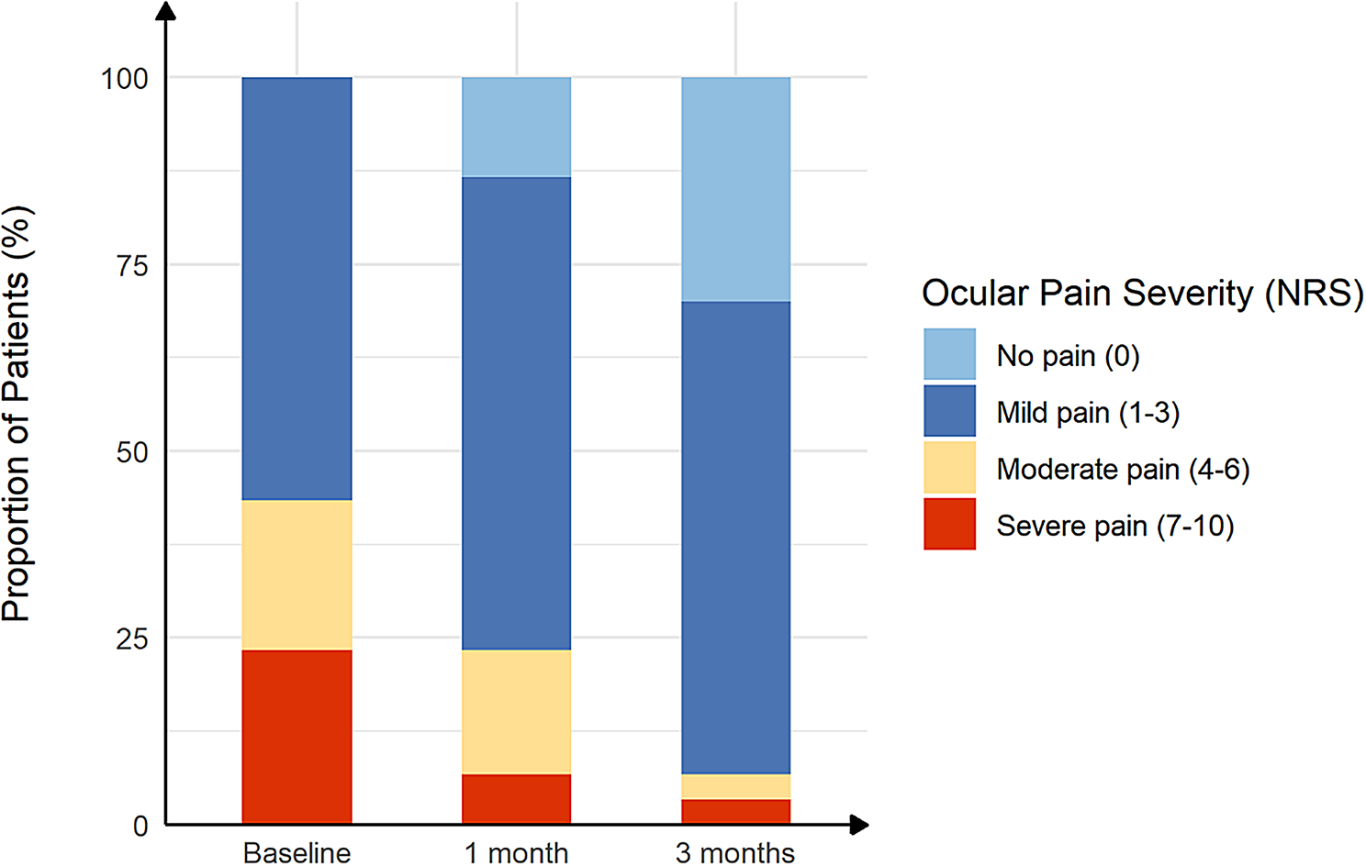
The proportion of ocular pain severity in post–refractive surgery DED patients with ocular pain before and after treatment. The severity of ocular pain was scored according to Numerical Rating Scale (NRS) (range, 0–10)

Incorrect

**Fig. 3:**
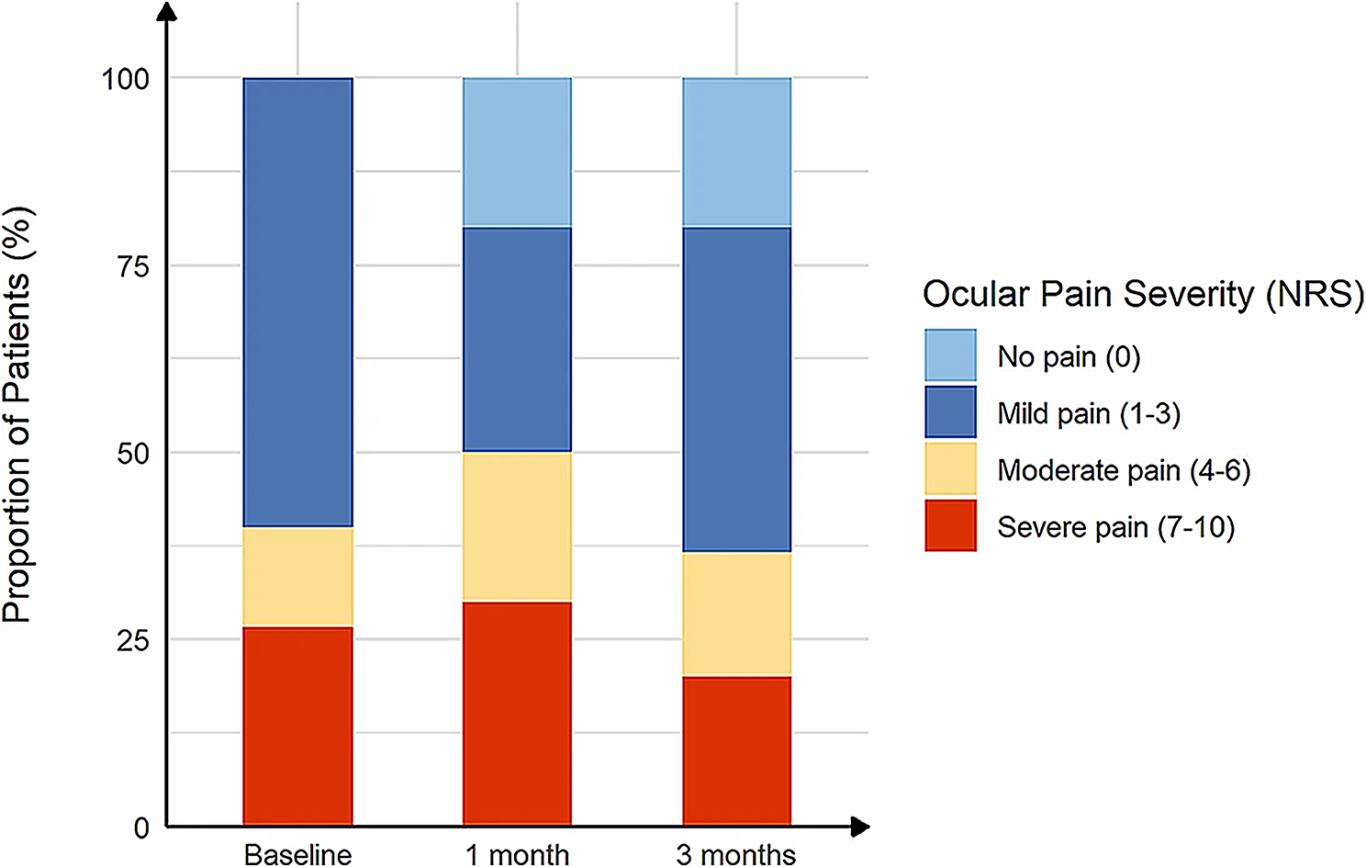
The proportion of ocular pain severity in post–refractive surgery DED patients with ocular pain before and after treatment. The severity of ocular pain was scored according to Numerical Rating Scale (NRS) (range, 0–10)

The original article has been updated.
